# Cannabidiol at Nanomolar Concentrations Negatively Affects Signaling through the Adenosine A_2A_ Receptor

**DOI:** 10.3390/ijms242417500

**Published:** 2023-12-15

**Authors:** Iu Raïch, Jaume Lillo, Carlos Ferreiro-Vera, Verónica Sánchez de Medina, Gemma Navarro, Rafael Franco

**Affiliations:** 1 Department of Biochemistry and Physiology, School of Pharmacy and Food Science, Universitat de Barcelona, 08028 Barcelona, Spain; iuraichipanisello@gmail.com (I.R.); g.navarro@ub.edu (G.N.); 2 CiberNed, Network Center for Neurodegenerative Diseases, Spanish National Health Institute Carlos III, 28029 Madrid, Spain; jaumelillo@ub.edu; 3 Department of Biochemistry and Molecular Biomedicine, School of Biology, Universitat de Barcelona, 08028 Barcelona, Spain; 4 Phytoplant Research S.L.U., 14014 Córdoba, Spain; c.ferreiro@phytoplant.es (C.F.-V.); v.sanchez@phytoplant.es (V.S.d.M.); 5 Institute of Neurosciences, Universitat de Barcelona, 08007 Barcelona, Spain; 6 School of Chemistry, Universitat de Barcelona, 08028 Barcelona, Spain

**Keywords:** MAPK, ERK phosphorylation, cAMP, cannabinoids, cannabinoid receptors, adenosine receptors

## Abstract

Cannabidiol (CBD) is a phytocannabinoid with potential as a therapy for a variety of diseases. CBD may act via cannabinoid receptors but also via other G-protein-coupled receptors (GPCRs), including the adenosine A_2A_ receptor. Homogenous binding and signaling assays in Chinese hamster ovary (CHO) cells expressing the human version of the A_2A_ receptor were performed to address the effect of CBD on receptor functionality. CBD was not able to compete for the binding of a SCH 442416 derivative labeled with a red emitting fluorescent probe that is a selective antagonist that binds to the orthosteric site of the receptor. However, CBD reduced the effect of the selective A_2A_ receptor agonist, CGS 21680, on G_s_-coupling and on the activation of the mitogen activated kinase signaling pathway. It is suggested that CBD is a negative allosteric modulator of the A_2A_ receptor.

## 1. Introduction

Two of the compounds in *Cannabis Sativa* L., (−)-Δ^9^-trans-tetrahydrocannabinol (THC, CAS 1972-08-3), and (−)-trans-cannabidiol (CBD, CAS 13956-29-1), have been extensively studied in translational research. Both can interact with the receptor that mediates the psychotropic effect of some phytocannabinoids, namely the cannabinoid CB_1_ receptor, but whereas THC is psychotropic, CBD is not. Accordingly, CBD is attracting more attention as a potential therapeutic entity to combat a variety of diseases. 

CBD is already approved for human consumption; Epidiolex^TM^ is an oral solution of CBD that, according to the US Federal Drug Administration, is indicated for the “*treatment of seizures associated with Lennox*-*Gastaut syndrome or Dravet syndrome in patients 1 years of age and older. It has also approved Epidiolex for the treatment of seizures associated with tuberous sclerosis complex in patients 1 year of age or older*” (https://www.fda.gov/news-events/public-health-focus/fda-regulation-cannabis-and-cannabis-derived-products-including-cannabidiol-cbd; accessed on 25 August 2023).

Probably the first studies on the chemical structure of CBD were performed in 1940 [1,2]. To the best of our knowledge the complete elucidation of the structure and stereochemistry of CBD obtained from *Cannabis sativa* L., was achieved in 1963 by Mechoulam and Shvo [3]. The systematic name of the compound is 2-[(1R,6R)-3-Methyl-6-(prop-1-en-2-yl) cyclohex-2-en-1-yl]-5-pentylbenzene-1,3-diol, showing that it is an interesting structure that contains a cyclohexene and a benzene ring with two hydroxyls and a short aliphatic chain. Such a relatively small and compact structure with hydrophilic and hydrophobic components is prone to interact with lipids and proteins. In fact, several effects of CBD administration have been reported. Early reports showed alteration of slow-wave sleep latency and time occurring in rats at a relatively high dosage (20–40 mg/kg) [4] and a regulation of corticosteroid synthesis by mouse tumor cells [5]. One of the first suspected modes of action was the inhibition of testicular esterase isozymes in the synthesis of sex hormones [6]. Subsequent studies have made it possible to find effects of the phytocannabinoid in the periphery and in the central nervous system. In humans, CBD affects hemodynamics and has a lowering effect on blood pressure upon acute administration; further clinical trials are needed to assess longer-term cannabidiol usage in treated and untreated hypertension [7,8]. The mode of action on cardiovascular responses to acute restraint stress in rats involves serotonin 5-HT_1_ receptors [9]. The potential of CBD in combating the side effects derived from the hypoxia of the neonate is well supported by strong preclinical/translational data [10,11,12,13]. The underlying mechanism appears to involve two G-protein-coupled receptors, namely the cannabinoid CB_2_ and the serotonin 5-HT_1A_ [7,8,9]. In fact, CBD was long ago identified as an agonist of the 5-HT_1A_R (with low potency, in the micromolar range) [14]. Recently, Bosquez-Berger et al. have convincingly shown that CBD is an allosteric modulator of the µ-opioid receptor [15]. A list of potential receptor targets of CBD was provided in a 2015 review paper in which the A_2A_R was not included [16]. Effects of CBD, mainly beneficial, have been described in epilepsy, inflammation (including neuroinflammation), pain, malignancies and psychosis [17,18,19,20,21,22,23,24,25,26,27,28,29,30].

Upon the discovery of two cannabinoid receptors, CB_1_ and CB_2_, having 2-arachidonoyl glycerol and anandamide as endogenous agonists [31,32,33], the binding and action of the most studied phytocannabinoids, THC and CBD, was characterized in both animal models and in heterologous systems expressing CB_1_ or CB_2_ receptors. It is intriguing why THC acting on the CB_1_ receptor is psychotropic whereas other cannabinoids that have similar nanomolar affinity for the receptor are not; CBD does not have a high affinity for the orthosteric site of the CB_1_ receptor nor is it psychotropic [34,35]. One possible explanation is heterogeneity in the orthosteric site allowing a qualitative different binding mode depending on the chemical structure of the cannabinoid. In fact, The *K*_*i*_ value of several phytocannabinoids obtained in competition assays using membranes from cells expressing the CB_1_ receptor is very different when using [^3^H] CP 55940 than when using [^3^H] WIN 55212-2 [36]. Remarkably, CBD can interact with the two cannabinoid receptors, CB_1_ and CB_2_, in a dual manner. At high concentrations the compound may go to the orthosteric site of CB_1_ or CB_2_ receptors and act as an agonist. Competition assays using radiolabeled molecules targeting the orthosteric site in membranes from CHO cells expressing the human version of the CB_1_ or the CB_2_ receptors, show K_i_ values for CBD in the micromolar range [36,37]. In contrast, at nanomolar concentrations CBD may act as an allosteric modulator of cannabinoid receptors [37,38]. We have been able to identify the allosteric site on the CB_2_ receptor and design CBD analogues that can behave as negative or negative allosteric modulators [39]. Taken together, the data suggests that CBD is capable of binding to different proteins, both to the orthosteric site and to allosteric sites.

Ribeiro et al., showed in 2012 that the anti-inflammatory effect of cannabidiol administered to mice with acute lung injury was blocked by a selective A_2A_ receptor antagonist. The authors suggested that CBD could be exerting the anti-inflammatory action by increasing the concentration of adenosine and, consequently, the adenosinergic tone mediated by the A_2A_ receptor [40]. Another molecular mechanism by which CBD may regulate inflammation in an adenosine-receptor-dependent manner is by inhibiting a nucleoside transporter whose role is passive, i.e., the direction of the flux depends on the concentration of adenosine at both sides of the membrane [41]. Inhibition by CBD of equilibrative nucleoside transporters would alter the extracellular concentration of adenosine, which regulates immunological responses via activation of adenosine receptors present in the cells of the immune system [42,43,44,45,46,47].

Adenosine A_2A_ receptors show promise as therapeutic targets in a variety of diseases. Istradefylline, a drug that selectively antagonizes the receptor, has been approved in Japan and the US for adjuvant therapy of Parkinson’s disease [48,49,50]. Although istradefylline was approved for addressing symptoms, results in animal models suggest that antagonists of A_2A_ receptors may be neuroprotective [51,52,53,54,55,56,57,58,59]. Interestingly, the non-selective antagonists of adenosine receptors present in coffee and tea, caffeine and theophylline, reduce the risk of suffering from Parkinson’s or Alzheimer’s disease [60,61,62,63,64,65,66,67,68,69,70]. Recently, evidence has been gathered that points to the A_2A_ receptor as a mediator of caffeine’s potential in preventing Alzheimer’s disease [71]. In summary, reducing adenosinergic tone in the brain appears to be neuroprotective, and antagonizing the action of adenosine on the A_2A_ receptor is therapeutic in patients with neurodegenerative diseases.

In addition, interest on A_2A_ receptor antagonists is based in its potential to enhance the capability of cells of the immune system to kill cancer cells [72,73,74]. There are currently several clinical trials to assess the efficacy of adenosine A_2A_ (and of A_2B_) receptor antagonists for boosting anti-cancer immunotherapy [75]. Several years ago the laboratory of Sitkovsky and colleagues suggested that antagonists of the A_2A_ receptor could enhance the anti-tumor activity of the cells of the immune system [76]. Subsequent work in different laboratories has shown that antagonists of these two G_s_-coupled receptors could boost anti-cancer immunotherapeutic or chemotherapeutic interventions. There are ongoing clinical trials using A_2A_ or A_2B_ receptor antagonists and also dual A_2A_/A_2B_ antagonists that could lead to drug candidates for approval for cancer therapy in the near future (see [75] for a review). Reduction in adenosinergic tone may improve the efficacy of anticancer surveillance by A_2A_ receptor-expressing cells of the immune system.

The objective of this work was to determine in a heterologous expression system if CBD could be activating the A_2A_ receptor by interacting with the orthosteric site or if CBD could affect the effect of CGS 21680, a selective A_2A_ receptor agonist.

## 2. Results

### 2.1. Homogenous Binding Assays

The development of a non-radioactivity-based method for measuring ligand binding to membrane receptors, specifically to G-protein-coupled receptors, has led to several advantages, both technical and conceptual. The burden of using radioactive ligands and removing radioactive waste is avoided, while binding can be measured in living cells rather than isolated membranes. It should be noted that radioligand binding is usually performed in heterogenous mixtures composed of both plasma membrane fragments and intracellular membrane fragments. In addition, the non-radioactive methods used here are homogenous, i.e., no washing and/or centrifugations steps are required. Homogenous Time-Resolved Fluorescence (HTRF) binding assays were performed in living cells expressing the Halo-A_2A_ receptor.

**Figure 1:**
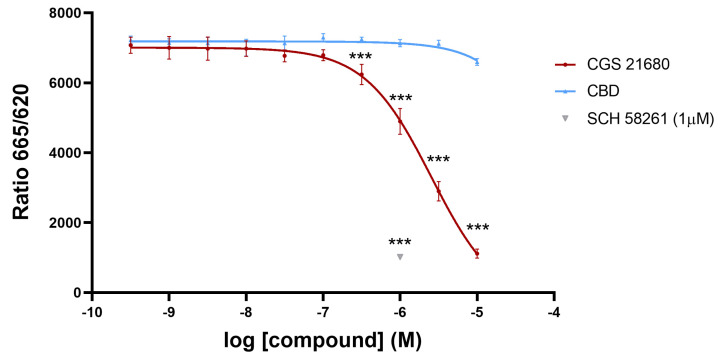
HTRF-based assays. Competition curve of specific binding of 20 nM SCH 442416 derivative labeled with a red emitting HTRF fluorescent with increasing concentrations of CGS 21680 or CBD. The single point in grey corresponds to the ratio corresponding to 20 nM labeled receptor ligand in the presence of 1 µM SCH 58261, a selective A_2A_ receptor antagonist (this value serves to define which is the maximal reduction in ratio achieved by a selective competitor). Data are the mean ± SEM of a representative experiment (*n* = 4). HTRF ratio = 665 nm acceptor signal/620 nm donor signal ×10,000. Inferential statistical analysis was conducted using the Student’s *t*-test to assess significant differences between groups. *** *p* < 0.001 versus CBD condition.

First, we assessed whether CBD could be interacting with the orthosteric site of the A_2A_ receptor. The assays were based on the recently developed Halo/tag technology that allows measuring ligand binding to receptors in living cells and without the requirement of radiolabeled compounds. Figure 1 shows competition by the selective A_2A_ receptor agonist, CGS 21680, of the binding of the SCH 442416 derivative labeled with a red emitting HTRF fluorescent probe to the A_2A_R expressed in CHO cells. Since the IC_50_ value of competition by CGS 21680 was relatively high, we double checked the specificity of the assay using a selective antagonist, SCH 58261, which completely competed the binding of the labeled probe to the same level that the highest concentration of CGS 21680 used. Once the assay was validated, we tested the CBD, which was unable to significantly compete for the binding of the labeled antagonist to the receptor. Therefore, under the assay conditions, CBD is not identified as an orthosteric ligand of the A_2A_R. Currently, demonstrating a CBD/A_2A_R interaction is technically challenging due to the lack of tritiated CBD for use in radioligand binding experiments and ad hoc probe-labeled CBD for performing HTRF assays.

### 2.2. Signaling Assays

**Figure 2:**
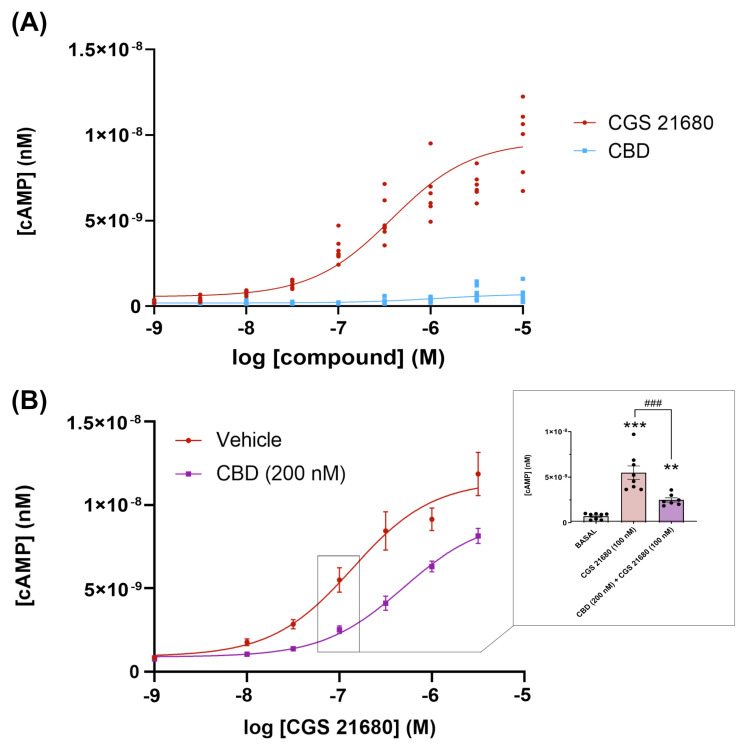
Gs-mediated signaling in CHO cells expressing the A_2A_ receptor. cAMP levels were determined as described in the Methods. (**A**). Concentration response–curves for CGS 21680 and CBD. Data points are from a representative experiment (each point with six replicates). Inferential statistical analysis was conducted using the Student’s *t*-test to assess significant differences. *** *p* < 0.001 versus CBD treatment. (**B**). Cells were pre-treated (15 min) with 200 nM CBD (purple) or ^cAMP^medium (red) and subsequently stimulated with different concentrations of CGS 21680. The inset shows the statistical analysis of data obtained for 100 nM CGS 21680 in the absence and presence of 200 nM CBD. For comparison the basal levels of cAMP are also shown (obtained by placing ^cAMP^medium with neither CGS 21680 nor CGS 21680 plus CBD). The values are the mean ± SEM of 3 different experiments (each point with three replicates). One-way ANOVA and Bonferroni’s multiple comparison post-hoc tests were used for statistical analysis. ** *p* < 0.05, *** *p* < 0.001 versus basal condition and ^###^ *p* < 0.001 versus CGS 21680 treatment.

Next, we assessed whether CBD could be acting as a protean agonist as defined by Kenakin [77], i.e., able to engage Gs-coupled A_2A_Rs and/or to reduce constitutive activity of the receptor without interacting with the orthosteric site. Engagement of G_s_ would in turn lead to activation of adenylyl cyclase and the subsequent increase in the intracellular levels of cAMP. The concentration–response curve of cAMP production in Figure 2A, shows that the cAMP production is negligible. Constitutive activity, if any, was negligible as per the low cAMP concentration (<1 nM) found in cells that were not treated with the selective agonist CGS 21680; in addition, CBD did not lead to any decrease in basal levels of cAMP. These results show that CBD is neither an orthosteric nor a protean agonist of the receptor. CBD was, however, able to regulate the response of the receptor to CGS 21680. The result was consistently found, and concentration–response curves such as the one displayed in Figure 2B showed that the concentration of the selective agonist, CGS 21680, providing half the maximum response (EC_50_) increased (3.5–5-fold range) in the presence of 200 nM CBD. In the representative graph in Figure 2B the EC_50_ value for CGS 21680 went from 143 nM in the absence of CBD to 650 nM in the presence of 200 nM CBD. Thus, the regulation was negative (decrease in potency), but, interestingly, the maximal effect exerted by CGS 21680 was not drastically reduced. To further investigate whether CBD could be a negative allosteric modulator of the A_2A_R, experiments were performed in CHO cells expressing or not expressing the A_2A_R and incubated with 500 nM forskolin, an activator of adenylate cyclase. Treatment of these cells with 200 nM or 1 µM CBD did not produce any significant decrease in cAMP (Supplementary Figure S1), indicating that the phytocannabinoid was not acting directly on the enzymes that produce or degrade cAMP.

**Figure 3:**
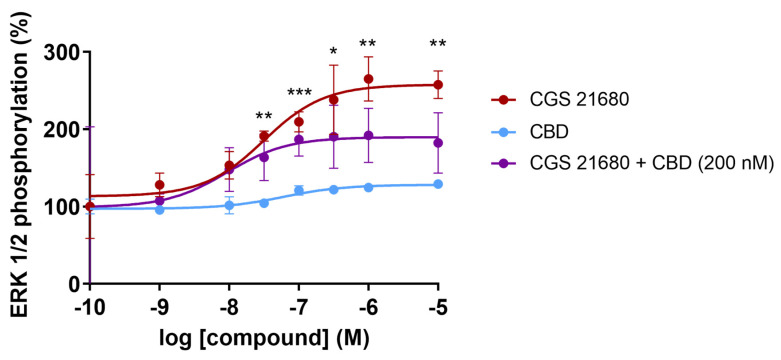
ERK1/2 phosphorylation in CHO cells expressing the A_2A_ receptor. Cells were pre-treated with DMEM (red) or 200 nM CBD prepared in DMEM (purple) before stimulation with the selective A_2A_ receptor agonist, CGS 21680. The effect of CBD at different concentrations was also tested (blue). Data are expressed in percentage over pERK1/2 values obtained adding 30 µL DMEM (basal) instead of 30 µL of DMEM containing CGS 21680, CBD or both. Values are the mean ± SEM. Data from a representative experiment of 4 carried out are shown. Inferential statistical analysis was conducted using the Student’s *t*-test to assess significant differences between groups. * *p* < 0.05, ** *p* < 0.01 and *** *p* < 0.001 versus CBD condition.

Finally, we checked whether CBD could regulate the MAPK pathway activation caused by CGS 21680 treatment of A_2A_ receptor-expressing CHO cells. The link of adenosine receptor to the MAPK signaling pathway was reported in late 1990s [78,79]. It was reported that CGS 21680 leads to a concentration-dependent increase in the degree of activation of the MAPK pathway measured by immunoblotting using an affinity-purified rabbit polyclonal antibody that recognized the phosphorylated forms of ERK (pERK1 and pERK2). The increase in the degree of ERK phosphorylation occurred in HEK-293 and in CHO cells expressing the A_2A_R, but also in human umbilical vein endothelial cells, which endogenously express the receptor [78]. The concentration–response curve for the selective agonist, CGS 21680, shows a significant increase in the level of phosphorylated ERKs with a potency in the nanomolar range (EC_50_ = 31 nM). In contrast, the effect of CBD was negligible at all concentrations tested. However, CBD at a concentration of 200 nM was able to markedly decrease the degree of activation of the pathway by approximately 40% (Figure 3). Interestingly, the potency of CGS 21680 did not increase in the presence of 200 nM CBD; in the representative graph in Figure 3, CBD did not cause an increase but a decrease in the EC_50_ value for the agonist (from 28 nM in the absence of CBD to 10 nM in the presence of 200 nM CBD).

## 3. Discussion

Allosteric modulators are molecules that bind to a site on a protein other than the protein’s active site, and by doing so, they can modify the protein’s activity [80]. Due to difficulties in the approval of new drugs targeting the orthosteric sites of G-protein-coupled receptors, mainly due to side effects, allosteric modulators are emerging as good alternatives in drug discovery. For instance, allosteric modulators can provide higher specificity and selectivity for a particular GPCR. Some G-protein-coupled receptors have similar structures and can have overlapping functions, so allosteric modulators can help target a specific subtype without affecting others. Alternatively, a given allosteric modulator can provide benefits by affecting different G-protein-coupled receptors. Allosteric modulators do not compete with endogenous ligands for the receptor’s active site. This means that allosteric modulators can work in conjunction with the endogenous agonists, potentially providing positive/synergistic or negative/antagonistic effects [81,82,83,84]. Indeed, such molecules can fine-tune the signaling and/or lead to partial activation or inhibition of the receptor, which can be therapeutically advantageous, especially for conditions where full activation or inhibition might lead to unwanted side effects. Other advantages are related to the mitigation of desensitization and tolerance, which is one of the possible drawbacks derived from the use of orthosteric compounds as therapeutic drugs. The potential of allosteric modulators in drug discovery also stands for an enhanced safety profile; the risk of adverse effects is reduced by avoiding the excessive activation or blockade when using, respectively, orthosteric agonist or orthosteric antagonists. In summary, the benefits of allosteric modulators for GPCRs include enhanced specificity, fine-tuning of signaling, reduced side effects and the potential for innovative therapeutic strategies. The main issue is that the design and identification of suitable allosteric molecules is challenging. The 3D structure, available for several G-protein-coupled receptors, can undoubtedly help [85]. Also of interest is the possibility of preparing molecules targeting the interacting surfaces in dimers of G-protein-coupled receptors [86,87].

The present study was prompted by the notion that CBD could be an allosteric modulator of adenosine receptors. To the best of our knowledge this notion comes from the report of CBD actions in a murine model of acute lung injury. In this in vivo model, administered CBD had an anti-inflammatory effect through a mechanism that appears to involve the A_2A_ receptor [40]. The anti-inflammatory effect of CBD was attributed to an increase in adenosine acting on A_2A_ receptors because it is reversed by an antagonist of the adenosine receptor. This possibility contrasts with the results obtained here in the heterologous expression system because CBD is neither acting as an agonist nor improving the effect of CGS 21680, a selective A_2A_ receptor agonist. In fact, our results suggest that CBD acts as a negative allosteric modulator of the A_2A_ receptor. In a report published in August 2023 [88], the negative modulation of A_2A_ receptor signaling by CBD is shown using a methodology to assess signaling that is different from the one used here. The authors used a BRET-based assay to measure the effect of CBD upon the binding of MRS7396, a bitopic compound that interacts with the A_2A_ receptor [89]. Their results lead to the same conclusions as ours, i.e., that CBD does not interact with the orthosteric site of the A_2A_ receptor. The simplest, Ockham’s razor interpretation of the results is that CBD is allosterically modulating the receptor. Considering such a possibility, the binding of CBD to the allosteric site would lead to conformational changes that would affect signaling but not the binding of antagonists to the orthosteric site.

From a medicinal chemistry point of view, it is interesting that CBD is (i) affecting the functionality of different G-protein-coupled receptors and (ii) interacting with orthosteric and allosteric sites in cannabinoid CB_1_ and CB_2_ receptors [37,38]. The resolution of the 3D structure of the CB_2_ receptor [90,91,92] and the development of homobivalent ligands of the same receptor [93] has increased the knowledge on how CBD may interact with two different sites in the same receptor. Homobivalent ligands of the CB_2_R were designed considering the atypical position of the orthosteric site of lipid G-protein-coupled receptors, which is not open to the extracellular milieu; agonists reach the orthosteric site through a narrow entrance that opens into the cell-membrane lipid bilayer [94]. Subsequently, it was possible to synthesize both positive and negative allosteric modulators of the CB_2_R by considering a potential allosteric site located at the entrance of the orthosteric site; more precisely, in the hydrophobic pocket between transmembrane domains 2, 3 and 7 of the CB_2_R [39]. Since the structure of the adenosine receptor is known [95,96,97], future work should address, using A_2A_ receptor mutants, which amino acids and which transmembrane domains are involved in the CBD-A_2A_ receptor interaction.

It is intriguing that CBD has so many possibilities of interaction with G-protein-coupled receptors; both at the orthosteric site, as in the case of cannabinoid and serotonin 5-HT_1A_ [7,8,9] receptors, and at allosteric sites, as in the case of cannabinoid and A_2A_ receptors. Consequently, future work should also address whether (i) there is a common motif in different G-protein-coupled receptors that allows CBD to act as an allosteric effector and (ii) the structure of the allosteric site is, to some extent, like the orthosteric site of cannabinoid and serotonin 5-HT_1A_ receptors. Another possibility cannot be ruled out, which is that CBD affects the properties of the lipid bilayer in such a way that the functionality of the GPCR is indirectly affected.

## 4. Materials and Methods

### 4.1. Reagents

CGS 21680 (3-{4-[2-({6-Amino-9-[(2R,3R,4S,5S)-5-(ethylcarbamoyl)-3,4-dihydroxytetrahydro-2-furanyl]-9H-purin-2-yl} amino) ethyl] phenyl}propanoic acid) and SCH 58261 (2-(2-Furanyl)-7-(2-phenylethyl)-7H- pyrazolo[4,3-e][1,2,4] triazolo[1,5-c] pyrimidin-5-amine) were purchased from Tocris Biosciences (Bristol, UK). CBD was purified by Phytoplant Research S.L.U. from a GOYA variety following a direct crystallization method that is described elsewhere [98]. The purity was set at >98% (data provided by Phytoplant Research S.L.U., Cordoba, Spain). HEPES (2-[4-(2-hydroxyethyl) piperazin-1-yl] ethane-1-sulfonic acid) was purchased from Sigma Aldrich (St. Louis, MO, USA). Stock solutions were prepared in dimethyl sulfoxide (DMSO). Aliquots of these stock solutions were kept frozen at 20 °C until use. In all cases, CGS 21680, SCH 58261 and CBD, the concentration in each aliquot was 10 mM in pure DMSO. At the highest concentration of CGS 21680, the SCH 58261 and CBD used in the DMSO concentration was 0.1%; this amount of DMSO does not provoke any significant effect in cAMP intracellular levels or in ERK1/2 phosphorylation levels. 

### 4.2. Cell Culture and Transient Transfection

Chinese hamster ovary cells (CHO) were grown in Dulbecco’s modified Eagle’s medium (Gibco, Paisley, Scotland, UK) supplemented with 1 mM sodium pyruvate, 2 mM L-glutamine, MEM nonessential amino acids solution (1/100), 100 units/mL penicillin/streptomycin and 10% (*v*/*v*) heat inactivated fetal bovine serum (FBS) (all supplements were from Invitrogen, (Paisley, Scotland, UK)). Cells were cultured in a humid atmosphere of 5% CO_2_ at 37 °C. Seeding was performed in either 6-well (for cAMP determination) or 96-well (for ERK phosphorylation assay) plates. After 24 h in culture, cells were transiently transfected with cDNA coding for the wild-type A_2A_ receptor. Polyethyleneimine (PEI) is a commonly used cationic polymer for transfection, which is the process of introducing foreign genetic material, such as plasmid DNA or RNA, into cells. PEI can form complexes with negatively charged nucleic acids and facilitate their uptake into cells. The method used, described elsewhere [99,100], consists of the formation of PEI-DNA complexes by diluting in serum-free medium at a concentration of 2 µg PEI per 1 µg DNA. The plasmid at the indicated concentration was mixed with PEI solution by gently pipetting. Incubation was performed at room temperature for 10 min to allow complex formation. The culture medium of cells plated 24 h earlier, so that they were approximately 70–80% confluent at the time of transfection, was replaced by fresh medium to improve transfection efficiency. The PEI-DNA complex mixture was added to cells dropwise, and the plate was swirled gently to ensure even distribution. Cells were incubated at 37 °C in a humid atmosphere of 5% CO_2_ for 4–6 h. After incubation, the transfection medium was removed and replaced with complete culture medium, and assays were performed after 48 h incubation at 37 °C in a 5% CO_2,_ humid atmosphere. Transfection efficiency was >70%. The sequences in the plasmids were those coding for human receptors. It should be noted that within a given experimental session, for instance the determination of cAMP levels, all agonists were tested in the same batch of transfected cells.

### 4.3. Homogeneous Time-Resolved Fluorescence (HTRF) Binding Assay

Homogeneous Time-Resolved Fluorescence (HTRF) is instrumental in biochemical and cell-based assays to study molecular interactions, including protein–protein interactions and receptor–ligand binding. When combined with the HaloTag technology, HTRF offers a powerful and sensitive method to investigate various biomolecular interactions in a homogeneous format, meaning that separation steps are not required. The pHalo is a plasmid vector containing the gene encoding the HaloTag protein, which can be used for creating fusion proteins in cells. Ligand binding assays involving the HaloTag requires the covalent binding of a FRET donor, terbium (Tb) in the present method, and the use of a SCH 442416 derivative labeled with a red emitting HTRF fluorescent probe (SCH 442416 is a selective A_2A_R antagonist; probe attachment does not modify ligand selectivity but does slightly modify ligand–receptor affinity; https://www.cisbio.eu/adenosine-a2ar-fluorescent-probe-40263, accessed on 2 December 2023). The HaloTag-based assay is versatile and allows for sensitive and specific detection of ligand–receptor binding events. The covalent nature of the binding ensures stability and minimizes false positives.

CHO cells growing in 6-well plates were transiently transfected with 1.5 μg of plasmid encoding for the Halo-tagged human A_2A_ receptor (using pHalo-A_2A_R plasmid, Cisbio Bioassays, Codolet, France) and incubated at 37 °C in a 5% CO_2_ humid atmosphere (24 h).

At 48 h post-transfection, the culture medium was removed, cells were washed with PBS and incubated with 100 nM of Halo-Lumi4Tb, a terbium derivative of O6-benzylguanine (SSNPTBC, Cisbio Bioassays, Codolet, France) previously diluted in TagLite buffer (LABMED, Cisbio Bioassays, Codolet, France) for 1 h at 37 °C under a 5% CO_2_ humid atmosphere. After that, cells were washed with PBS to remove the excess of Halo-Lumi4Tb, detached with Versene (Gibco Life Technologies, Paisley, Scotland, UK), centrifuged at 1500 rpm for 5 min and resuspended in TagLite buffer. Densities of 10,000 cells/well were used to carry out binding assays in white opaque 384-well plates.

### 4.4. Non-Radioactive Homogeneous Time-Resolved FRET-Based Binding Assays

Saturation binding experiments were performed by incubating cells expressing Tb-labeled Halo-A_2A_R with increasing concentrations of a SCH 442416 derivative labeled with a red emitting HTRF fluorescent probe (purchased from Cisbio Bioassays, Codolet, France; https://www.cisbio.eu/adenosine-a2ar-fluorescent-probe-40263, accessed on 2 December 2023). The unspecific signal was obtained by incubating cells expressing Tb-labeled Halo-A_2A_ receptor with 25 μM CGS 21680 in the presence of 20 nM A_2A_ receptor ligand labeled with the HTRF fluorescent probe. Both, labeled SCH 442416 and unlabeled compounds, were diluted in TagLite buffer. For competition binding assays, CHO cells transiently expressing Tb-labeled Halo-A_2A_R were incubated with 20 nM fluorophore-conjugated A_2A_ receptor ligand in the presence of increasing concentrations (0–10 μM range) of CGS 21680 or CBD (or antagonist, SCH 58261, where indicated). Plates contained 10 μL of labeled cells, and 5 μL of tested compounds were added prior to the addition of 5 μL labeled A_2A_ receptor antagonist. Plates were then incubated for at least 2 h at room temperature prior to TR-FRET signal detection. A detailed description of Homogenous Time-Resolved Fluorescence assays is found elsewhere [101].

### 4.5. Signal Detection and Data Analysis

Donor emission (xenon flash lamp excitation; 10 flashes/well frequency) and determination of acceptor fluorescence emission was achieved using a microplate reader equipped with a FRET optic module (PHERAstar FS equipment; BMG Lab technologies, Offenburg, Germany). Excitation wavelength was 337; emission fluorescence was collected at 665 and 620 nm using the following time-resolved settings: delay, 150 ms; integration time, 500 ms. HTRF ratios were obtained by dividing the acceptor signal (665 nm) by the donor signal (620 nm) and multiplying this value by 10,000. The 10,000 multiplying factor is used solely for the purpose of easier data handling. Data were analyzed using Prism 8 software (GraphPad Software, Inc., San Diego, CA, USA). K_D_ (dissociation constant) = 12 nM. Note that B_max_ values obtained from Homogeneous Time-Resolved Fluorescence (HTRF) saturation curves do not reflect absolute values of receptor binding sites. K_i_ values were determined from competition binding assays by using the calculated IC_50_ and K_D_ values and the Cheng-Prusoff equation.

### 4.6. cAMP Determination

As the A_2A_ receptor couples to G_s_/G_olf_ proteins, its activation by agonists leads to increases in the intracellular concentration of adenosine cyclic 3′,5′-monophosphate (cAMP). The concentration of this first messenger was determined using a homogenous assay and using a calibration curve with solutions of cAMP at different concentrations (dynamic range between 10^−10^ and 10^−8^ M)

The Lance Ultra cAMP kit (Perkin Elmer, Waltham, MA, USA) was used for homogenous cAMP determination assay in living cells. The method consists of a time-resolved fluorescence resonance energy transfer (TR-FRET) immunoassay in which endogenous cAMP competes with europium (Eu) chelate-labeled cAMP tracer for binding sites on a cAMP-specific antibody labeled with the ULight^TM^ dye (Perkin Elmer). Light pulses at 320 nm excite the Eu of the tracer. The energy emitted by the excited Eu is transferred by FRET to ULight molecules on the antibodies, which in turn emit light at 665 nm. In the absence of cAMP, a maximal TR-FRET signal is achieved; when an agonist leads to an increase in cytosolic cAMP levels the competition between the unlabeled and the Eu-labeled cAMP species leads to a decrease in the TR-FRET signal, the emission fluorescence remains unmodified when equilibrium is achieved. cAMP concentrations per cell or per mg protein were determined using a standard curve using pure unlabeled cAMP. Residual energy from the Eu chelate will produce light at 615 nm.

CHO cells growing in 6-well plates were transiently transfected with cDNAs for A_2A_ receptors as described in Section 4.2. Forty-eight hours post-transfection and culturing in 6-well plates, the medium was replaced by serum-free Dulbecco’s Modified Eagle’s medium. Two hours later, cells were detached, isolated by centrifugation (5 min at 1500 rpm) and resuspended in ^cAMP^medium, which consists of Dulbecco’s Modified Eagle’s medium containing 5 mM HEPES (pH 7.4) and 50 mM zardaverine (phosphodiesterase inhibitor that prevents degradation of cAMP). Determination was performed in 384-well plates (Perkin Elmer) using 4000 cells/well. Cells were pretreated with 2 µL of 300 nM CBD or 2 µL of ^cAMP^medium for 15 min. Then cells were incubated for 15 min with 2 µL of ^cAMP^medium (to determine the basal levels of cAMP) or with 2 µL of ligands prepared in ^cAMP^medium. Fifteen minutes later, cAMP-Europium (cAMP-Eu) (5 µL) and fluorescent antibody (5 µL) were added. Incubation was prolonged for 1 h at 25 °C and the PHERAstar Flagship reader equipped with an HTRF optical module (BMG Lab technologies, Offenburg, Germany) was used for measuring the 665/620 ratio.

### 4.7. ERK1/2 Phosphorylation Assays

The link to the mitogen-activated kinase (MAPK) signaling pathway was assessed by a method that is homogeneous, avoids immunoblotting and directly measures levels of phosphorylated proteins in a cell-based format measuring (Alpha Screen SureFire kit; Perkin Elmer, Waltham, MA, USA). The kit contains an antibody that is specific for a phospho-epitope and another that is specific for another region (distal to the phospho-epitope) of extracellular signal-regulated kinase 1 and 2 (ERK1/2). One of the antibodies is biotinylated and binds to streptavidin-conjugated donor beads, and the second binds to protein A Sepharose beads that contain an acceptor. Only immuno-complexes that contain both antibodies can bind both beads and, therefore, donor to acceptor energy transfer can occur. Emission fluorescence is measured using an EnSpire Multimode Plate Reader (Perkin Elmer, Waltham, MA, USA). The kit uses the AlphaScreen^®^ technology that is based on the emission of singlet oxygen by the donor beads (which contain phthalocyanine, excited by the red light at 680 nm), which can diffuse to reach the acceptor beads where the energy of singlet oxygen is used by a cascade (thioxene–anthracine–rubrene), leading to the emission of light at a shorter wavelength (520–620 nm range) than the excitation light. Transparent Biocat Poly-D Lysine 96-well plates (Deltalab, Rubí, Spain) were used for cell culture. CHO cells were transiently transfected with cDNAs for A_2A_ receptor as described in Section 4.2. Forty-eight hours post-transfection, the medium was replaced by serum free Dulbecco’s Modified Eagle’s medium (DMEM). Incubation with 30 µL reagents (prepared in DMEM) or with 30 µL DMEM was prolonged for 10 min at 25 °C. PBS was used for a wash prior to treatment with lysis buffer (30 µL/well; Perkin Elmer, Waltham, MA, USA). Lysis was allowed for 15 min at 25 °C taking advantage of a shaker (Heidolph Titramax 100). 10 µL of each sample were dispensed into 384-well microplates (white ProxiPlate; Perkin Elmer; Waltham, MA, USA). Subsequently, 5 μL of beads containing the acceptor were added to each well and, after incubation in the dark for 2 h at 25 °C, 5 μL of beads containing the donor were added to each well. After 2 h incubation in the dark, fluorescence was determined. The effect of ligands was given in percentage respective to the reference value (basal). The value achieved in the absence of any treatment (30 µL DMEM) was taken as reference (basal = 100).

### 4.8. Data Handling and Statistical Analysis

Data related to experiments performed in the presence and/or absence of CBD were analyzed blindly. Data are presented as the mean ± SEM. Statistical analysis was performed with GraphPad Prism 9 software. In bar graphs, significance was analyzed by one-way ANOVA followed by Bonferroni’s multiple comparison post hoc test. In dose-response assays, inferential statistical analysis was performed using the Student’s *t*-test to asses significant differences between groups. Significance was considered when *p* < 0.05.

## 5. Conclusions

The aim of the present study was to address the question of whether CBD is affecting the functionality of the adenosine A_2A_ receptor by a direct mechanism. For this purpose, ligand binding and signaling assays were performed in a heterologous system expressing the human version of the receptor. A novel non-radioactive homogeneous assay was used to show that, in the assay conditions, we could not detect that CBD was affecting the binding of an orthosteric ligand to the receptor. Homogenous assays were also used to measure cAMP levels and the degree of ERK1/2 phosphorylation. The results demonstrate that CBD does not activate the A_2A_ receptor per se, but rather affects the signaling of the selective agonist, CGS 21680. These results suggest that CBD is a negative allosteric modulator of the receptor.

## Data Availability

Raw data are available from the corresponding author upon appropriate request.

## Supplementary Materials

The following supporting information can be downloaded at: https://www.mdpi.com/article/10.3390/ijms242417500/s1.
