# Supplementary material for: Cannabidiol at Nanomolar Concentrations Negatively Affects Signaling through the Adenosine A2A Receptor
Source: Int J Mol Sci. 2023 Dec 15;24(24):17500. doi: 10.3390/ijms242417500 (PMC10744210; doi:10.3390/ijms242417500)
Supplement: Supplementary file 1 [file ijms-24-17500-s001.zip › ijms-2715225-supplementary.pdf]

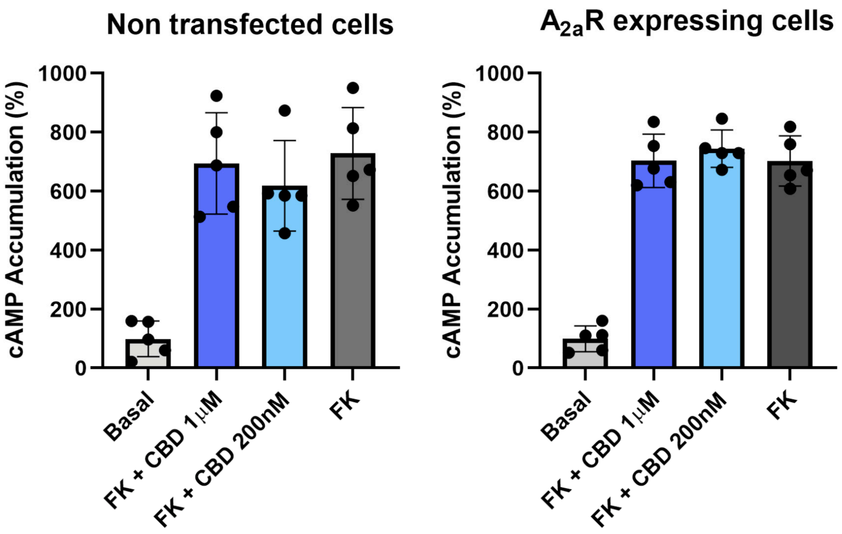

Supplementary Figure S1. Determination of cAMP levels in CHO cells treated with forskolin. Untransfected cells (panel A) or A2AR-expressing cells (panel B) were treated with 500 nM forskolin (FK) and with either cAMPmedium or CBD at two concentrations (200 nM and 1 μM; prepared in cAMPmedium). A representative experiment is shown in which the data are expressed as a percentage ± SD of the effect of 500 nM forskolin compared with the basal condition (100%). Data points from a representative experiment. In three different experiments and after statistical analysis using a one-way ANOVA followed by Bonferroni's post-hoc multiple comparison tests (versus forskolin–FK condition), no significant differences were found due to the presence of CBD.
